# Predictors of Entering a Hearing Aid Evaluation Period: A Prospective Study in Older Hearing-Help Seekers

**DOI:** 10.1177/2331216517744915

**Published:** 2017-12-14

**Authors:** Marieke Pronk, Dorly J.H. Deeg, Niek J. Versfeld, Martijn W. Heymans, Graham Naylor, Sophia E. Kramer

**Affiliations:** 1 Department of Otolaryngology–Head and Neck Surgery, section Ear & Hearing, Amsterdam Public Health research institute, VU University Medical Center, Amsterdam, The Netherlands; 2 Department of Epidemiology and Biostatistics, Amsterdam Public Health research institute, VU University Medical Center, Amsterdam, The Netherlands; 3 MRC/CSO Institute of Hearing Research (Part of the University of Nottingham), Scottish Section, Glasgow, UK

**Keywords:** hearing help-seeking, hearing aids, older adults, health belief model, stages of change

## Abstract

This study aimed to determine the predictors of entering a hearing aid evaluation period (HAEP) using a prospective design drawing on the health belief model and the transtheoretical model. In total, 377 older persons who presented with hearing problems to an Ear, Nose, and Throat specialist (*n* = 110) or a hearing aid dispenser (*n* = 267) filled in a baseline questionnaire. After 4 months, it was determined via a telephone interview whether or not participants had decided to enter a HAEP. Multivariable logistic regression analyses were applied to determine which baseline variables predicted HAEP status. A priori, candidate predictors were divided into ‘likely’ and ‘novel’ predictors based on the literature. The following variables turned out to be significant predictors: more expected hearing aid benefits, greater social pressure, and greater self-reported hearing disability. In addition, greater hearing loss severity and stigma were predictors in women but not in men. Of note, the predictive effect of self-reported hearing disability was modified by readiness such that with higher readiness, the positive predictive effect became stronger. None of the ‘novel’ predictors added significant predictive value. The results support the notion that predictors of hearing aid uptake are also predictive of entering a HAEP. This study shows that some of these predictors appear to be gender specific or are dependent on a person’s readiness for change. After assuring the external validity of the predictors, an important next step would be to develop prediction rules for use in clinical practice, so that older persons’ hearing help-seeking journey can be facilitated.

## Introduction

Hearing loss in older adults is often left untreated (Chia et al., 2007; Hartley, Rochtchina, Newall, Golding, & Mitchell, 2010), despite abundant evidence showing that hearing aid use improves communication and health outcomes (Chisolm et al., 2007; Mulrow, Tuley, & Aguilar, 1992). The factors associated with low hearing aid uptake that were reported in observational studies were summarized in two systematic reviews (Knudsen, Öberg, Nielsen, Naylor, & Kramer, 2010; Meyer & Hickson, 2012). For a number of factors, one or both reviews concluded that they significantly influenced uptake. In particular, a higher likelihood of hearing aid uptake was associated with older age, greater self-reported disability, and greater measured hearing loss (Knudsen et al., 2010; Meyer & Hickson, 2012). Furthermore, persons who acknowledged more benefits than barriers to hearing aid use, who had no or limited perceptions of hearing aid stigma, who experienced greater social support and greater social pressure to get a hearing aid, and whose family and friends had positive attitudes toward hearing aids were more likely to take up a hearing aid (Meyer & Hickson, 2012).

In both reviews, inconsistent or sparse evidence was found for many other factors. Examples include level of education, manual dexterity, coping style, technology use, cognitive functioning, and employment status (Knudsen et al., 2010; Meyer & Hickson, 2012). There were also factors that were judged as potentially important based on clinical experience or anecdotal evidence, such as patient–professional interaction (e.g., mismatches in views between clinicians and patients; Laplante-Lévesque et al., 2012) and self-efficacy of hearing aid handling.

Both Knudsen et al. (2010) and Meyer and Hickson (2012) mentioned two important caveats in the research performed so far. First, many of the included studies did not use appropriate multivariable prediction modeling. When following the successive steps of building a multivariable model (Moons, Altman, Reitsma, & Collins, 2015), negligibly weak predictors and confounders (i.e., factors without true predictive value) will be discarded from the model. This is in contrast to a univariate approach, in which these factors could—incorrectly—be considered relevant predictors, based on their univariate statistical significance. In addition, when the final multivariable model has been built, the unique predictive value of each of the included predictors is reflected because each predictor is adjusted for all other predictors with which they share variance. Second, all previous studies had a retrospective, cross-sectional design (i.e., uptake status and predictor factors were measured at the same moment in time). This approach hampered any strong conclusions about the factors’ true *predictive* value (see also Coulson, Ferguson, Henshaw, & Heffernan, 2016).

Since the publication of these two reviews, two observational studies have addressed the caveats outlined earlier. Meyer, Hickson, Lovelock, Lampert, and Khan (2014) retrospectively examined a wide range of audiological and nonaudiological factors in a sample of older Australian hearing aid candidates. Their multivariable model showed that the following factors were associated with hearing aid uptake: relatively positive attitudes toward hearing aids, high self-efficacy of hearing aid handling, high social support toward hearing aids, severe hearing loss, low cognitive reasoning skills, and receiving a pension. Saunders et al. (2016a) examined the predictive value of a smaller list of nonaudiological factors in older American veterans and were the first to apply a longitudinal design. Their multivariable model showed that greater baseline hearing loss severity and higher self-reported readiness for change significantly predicted uptake at 6 months’ follow-up.

In various countries, including the United States, Australia, and The Netherlands, a hearing aid trial period can be part of the hearing aid uptake process. In Australia, whether trials are offered varies from clinic to clinic, while in most states in the United States, a trial period is mandated. In general, during the trial period, clients can return the device for a full or partial refund of the costs. In contrast, in The Netherlands, every client has the right to enter a 2-month trial period *before* having to decide to purchase the hearing aid or not. This evaluation period is noncommittal and free of costs in all cases.

To the best of our knowledge, the predictors of entering a hearing aid trial period as a distinct outcome have never previously been analyzed. Understanding of such predictors is especially relevant for health-care systems (such as in The Netherlands) in which a trial period is a standard part of the purchase process, and precedes the purchase of the device. To distinguish the Dutch hearing aid trial period from trial periods in which the hearing aid has already been purchased, we henceforth refer to it as a hearing aid evaluation period (HAEP).

In first-time hearing aid users, it is plausible that the decision to enter a HAEP may be predominantly driven by prefitting expectations, while the decision to take up hearing aids may be driven by actual experiences with the device during a HAEP. Examples of factors for which expectations might differ from experience are as follows: hearing aid benefits, wearing comfort, self-efficacy of hearing aid handling, and monetary costs. The fact that expectations may differ from experience was recently supported by Saunders et al. (2016a). They found positive attitude changes in first-time help seekers who decided to take up a hearing aid, presumably as a consequence of their positive experiences with the devices. These changes did not occur in those who did not take one up.

**Figure 1.**
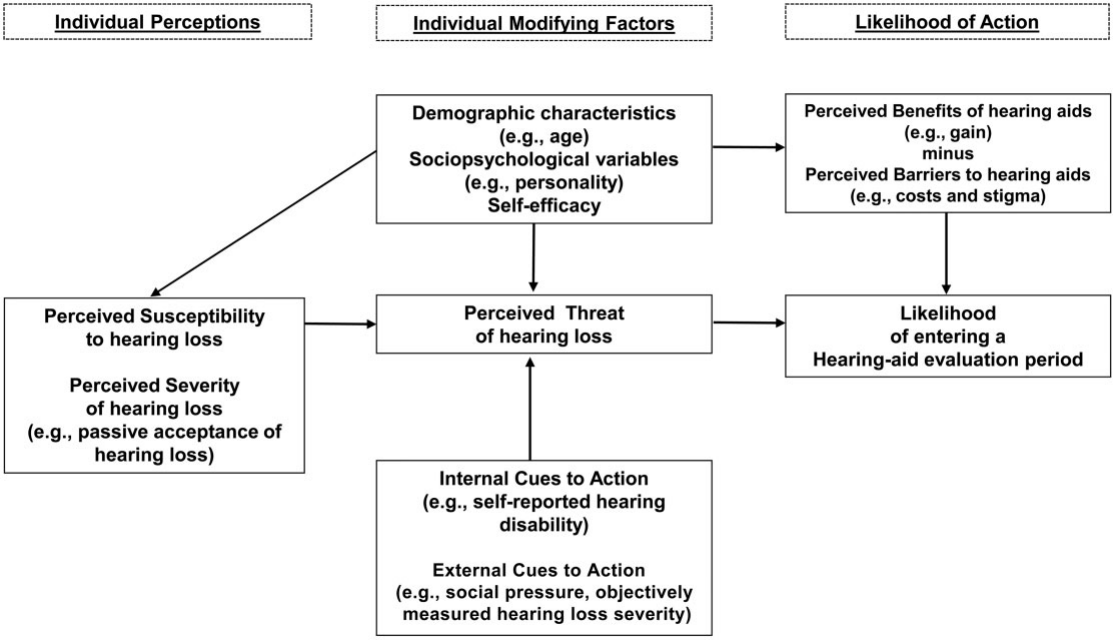
Schematic representation of constructs of the Health Belief Model along with examples of possible predictors of entering a hearing aid evaluation period. Adapted from Strecher and Rosenstock (1997).

Both Meyer et al. (2014) and Saunders et al. (2016a) incorporated the health belief model (HBM; Janz & Becker, 1984; Rosenstock, 1966) into their study designs. The HBM is a common health behavior change theory that can be used to identify the determinants of particular health-behavior changes and is increasingly applied to explain the hearing help-seeking process (Meyer et al., 2014; Saunders, Chisolm, & Wallhagen, 2012; Saunders, Frederick, Silverman, & Papesh, 2013; Saunders et al., 2016a, 2016b; Van den Brink, Wit, Kempen, & Van Heuvelen, 1996). When we apply the HBM to entering a HAEP (see Figure 1), a person would be more likely to enter a HAEP if he or she perceives high *severity* of the consequences of hearing loss and high *susceptibility* to developing a more severe hearing loss. These two factors together cover *perceived threat*. Also, a person who expects relatively many *benefits* (and few *barriers*) would be more likely to enter a HAEP. In addition, strong *internal cues to action* (e.g., self-reported hearing disability) and strong *external cues to action* (e.g., incentives by others, hearing loss as measured through the audiogram) would move people to enter a HAEP.

In the HBM, individual, modifying demographic and sociopsychological variables are defined. These can modify the effects that personal perceptions of *threat*, *benefits*, and *barriers* have on the health behavior change (see Figure 1). Examples of such variables are age, gender, personality, and self-efficacy of hearing aid use. Garstecki and Erler (1998) found that greater hearing loss severity in the better ear was significantly associated with uptake in women, while in men, only less denial and lower concerns over costs were significantly associated with uptake. In other words, they found supportive evidence that gender was a modifying variable for these factors. To our knowledge, no other researchers have examined whether there are individual factors that act as effect modifiers. Instead, individual factors (such as age and gender) were studied as *determinants* of uptake. We argue that it would be important to examine whether gender and age also modify the effects that predictors have on entering a HAEP. With increasing age, changes in societal participation (e.g., retirement) and health (e.g., more chronic diseases, more cognitive problems) occur (Comijs, Dik, Deeg, & Jonker, 2004; Ferrucci et al., 2016). We hypothesize that the predictive strength of factors like having paid work and self-reported hearing disability decrease with increasing age relative to factors like social leisure activity, comorbidity, and cognitive function (their strength would increase with increasing age).

In addition to age and gender, predictors of HAEP may be modified by a person’s degree of readiness to do something about their hearing. Readiness for behavior change originates from the stages construct of the transtheoretical stages of change model (TTM; Prochaska & DiClemente, 1983; Prochaska & Velicer, 1997). The TTM assumes that people progress via various stages toward adopting and maintaining a particular behavior change. For the purposes of this article, only the first three stages are relevant: (a) precontemplation (problem denial), (b) contemplation (problem awareness and ambivalence regarding the pros and cons of change), and (c) action (healthy behavior acquisition). Previous studies have shown that the stages are predictive of various hearing help-seeking outcomes (Ingo, Brännström, Andersson, Lunner, & Laplante-Lévesque, 2016; Laplante-Lévesque, Hickson, & Worrall, 2012, 2013; Saunders et al., 2013, 2016a). However, the question has never been studied whether the effects of certain predictors are different for persons with low intrinsic readiness (i.e., high precontemplation relative to contemplation and action) as opposed to persons with high intrinsic readiness (i.e., low in precontemplation relative to contemplation and action).

Taking the HBM and the TTM as guiding frameworks, the current study aims to identify the predictors of entering a HAEP in a large sample of older adults eligible for hearing aids who had never tried a hearing aid before. This was done by using a prospective study design, by including a wide range of candidate predictors, applying multivariate prediction modeling, and studying effect modification by age, gender, and readiness for change.

## Methods

### Sample and Procedures

The target population of this study comprises older adults without complex hearing problems, as defined in the Dutch field norm ‘Nationaal Overleg Audiologische Hulpmiddelen (NOAH)-4’ (NOAH, 2013). Roughly, all hearing losses except for presbyacusis are considered complex. Examples of complex hearing problems include psychosocial problems due to hearing loss, poor speech recognition in quiet, and bothersome tinnitus.

In The Netherlands, all hearing aids are obtained via a hearing aid dispenser (HAD). The official hearing aid prescription for older adults without complex hearing problems is carried out by Ear, Nose, and Throat (ENT) specialists, but this population is also allowed to directly visit a HAD for eligibility assessment and subsequent start of a HAEP. Whether a formal prescription by an ENT specialist is necessary depends on the requirements of the patient’s health insurance company.

In the HAD practice that participated in the study, care usually consisted of the following: a preparatory appointment (screening audiogram and assessment of interest regarding pursuit of a hearing aid), an intake appointment (speech audiometry and comprehensive pure tone audiometry; assessment of client needs; choice of a hearing aid), a fitting appointment, one or more fine-tuning appointments, and a purchase appointment (if applicable). The HAEP formally starts at the time of the fitting appointment. Usually, the cost of a hearing aid is partially (∼75%) reimbursed by a person’s health insurance if the pure tone average hearing threshold is 35 dB or greater, averaged across 1, 2, and 4 kHz in the ear to be aided. Therefore, the following inclusion criteria were applied for participant inclusion: self-initiated consultation with an ENT specialist or a HAD for a hearing loss assessment, absence of medical problems hindering hearing aid use (e.g., skin allergy), no prior hearing aid use, a minimum pure-tone average hearing threshold of 35 dB HL averaged across 1, 2, and 4 kHz in the ear(s) to be aided, and age 55 years or older.

Specialists from six ENT departments recruited participants. ENT patients were invited to participate in the study by their specialist at the end of their consultation. Patients who expressed interest in the study were phoned by the researchers 1 to 3 days later to further describe the study. Subsequently, a postal package was sent, consisting of a participant information letter, an informed consent form, a questionnaire, an instruction sheet to perform the telephone speech-in-noise test (see Candidate predictors), and a stamped return envelope. The research team recruited HAD clients originating from 118 HAD practices for the study. Clients were approached via a postal invitation shortly (around 3 to 5 days) after they had visited the HAD for their preparatory appointment. Their postal package was sent directly along with this invitation. We invited roughly equal numbers of clients who did and did not have an intake appointment scheduled, as we knew from the HAD that having an intake appointment scheduled was strongly associated with entering a HAEP. We thus attempted to recruit a sample with optimal spread in HAEP status. During recruitment, it was made clear to the participants that they did not need to know or disclose yet whether they wanted to pursue a hearing aid fitting. All participants were asked to fill out the questionnaire within 1 week of receipt and before their fitting appointment, if applicable. Four months after recruitment, participants were phoned and asked if they had entered a HAEP or not since baseline. Since there were no significant waiting lists, 4 months was considered sufficient to move from having been pronounced eligible for hearing aid fitting to entering a HAEP.

**Figure 2.**
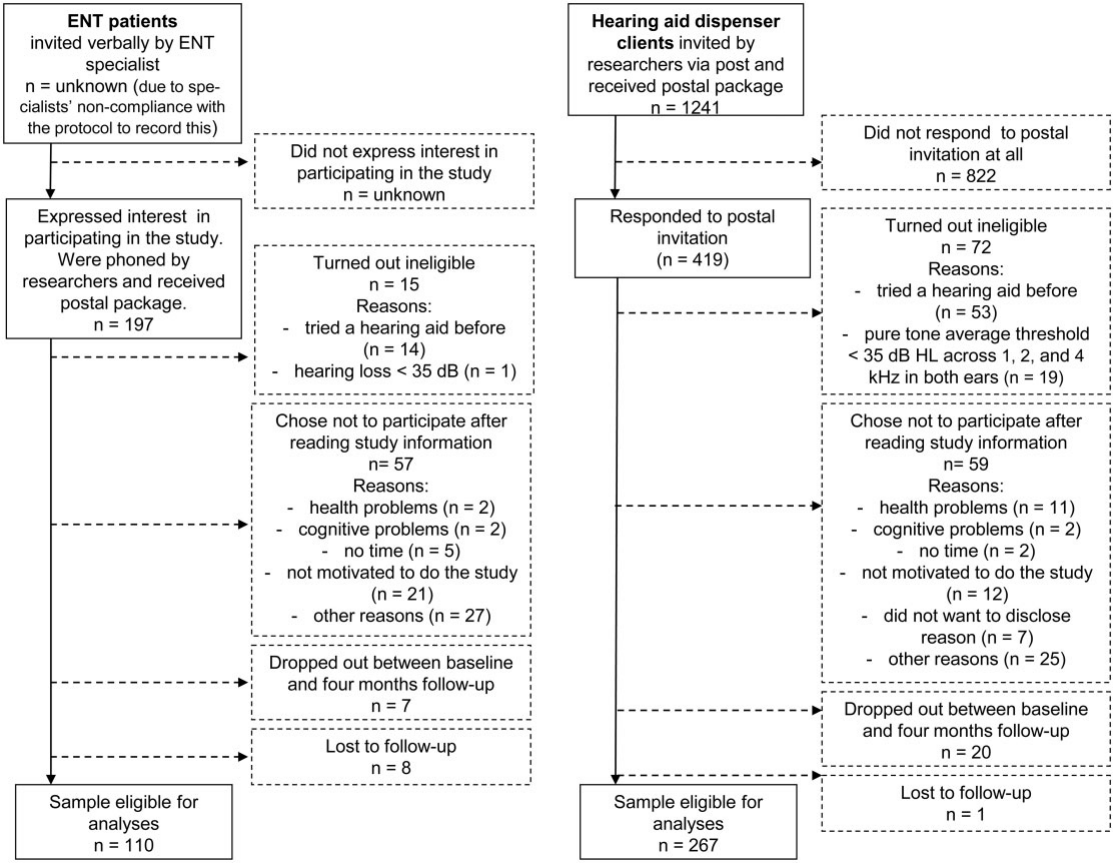
Participants’ flow through the study.

Figure 2 shows the participants’ flow through the study. The data of 110 ENT patients and 267 HAD clients could potentially be included in the analyses. Informed consent was obtained from all participants. Written approval for the study (name: PredictEAR, reference number: 2013.464) was obtained from the Dutch Institutional Review Board of the VU Medical University Center, Amsterdam (registered with the US Office for Human Research Protections as IRB00002991; FWA number: FWA00017598).

### Outcome Measure

HAEP status was defined as did or did not enter a HAEP between baseline and 4 months’ follow-up. Seven participants who had the hearing aid fitting appointment scheduled at the time of the follow-up interview but who had not been fitted yet were regarded as having entered a HAEP.

### Candidate Predictors

Given the conceptual overlap between hearing aid uptake and entering a HAEP as outcomes, the choice of the candidate predictors originates from the influencing factors for uptake as reviewed by Knudsen et al. (2010) and Meyer and Hickson (2012). Below, ‘likely’ and ‘novel’ candidate predictors are described. This categorization was used in the analyses (see Statistical Analyses section) and was based on the empirical evidence for the particular factor being associated with hearing aid uptake.

If one or both of the reviews concluded that there was strong evidence that a given factor influenced uptake, that factor was labeled a ‘likely’ predictor of entering a HAEP. In line with this, the following six predictors were regarded as ‘likely’ predictors: higher age, greater hearing loss severity, greater self-reported hearing disability, greater hearing aid stigma, and greater social pressure to get a hearing aid. The rationale for including each of the ‘likely’ predictors was already provided in the Introduction section. How the predictors were measured in the current study is described later.

For ‘novel’ predictors, the same reviews found that the evidence was sparse (i.e., only one study had shown a significant association with uptake), inconsistent (roughly equal numbers of studies showed significant associations and nonsignificant associations with uptake), or absent (no studies reported). The evidence found in the later studies by Saunders et al. (2016a) and Meyer et al. (2014) did not alter the categorization of ‘likely’ versus ‘novel’. For the ‘novel’ predictors, the rationale for including them in the current study is provided in the Appendix (see Supplementary Material). How the predictors were measured is described later.

#### Likely predictors

Unless stated otherwise, variables were collected via self-report (the postal questionnaire) and were analyzed as continuous variables.

– *Age* was included as age in years and was calculated from self-reported date of birth.
– *Hearing loss severity* was defined as the better-ear, pure-tone average hearing threshold (in dB HL) across 1, 2, and 4 kHz (3F-BEA). Audiograms were retrieved via ENT specialists or HADs. Air conduction hearing thresholds were obtained via a modified Hughson–Westlake procedure for octave frequencies between 250 and 8000 Hz.
– *Self-reported hearing disability* was measured using the Dutch, validated, 28-item version of the Amsterdam Inventory for Auditory Disability and Handicap (Kramer, Kapteyn, Festen, & Tobi, 1995; Boeschen Hospers et al., 2016). Scores can range from 0 to 74 (higher scores indicate more severe hearing disability). The scale showed an excellent internal consistency in our study sample (Cronbach’s α = .95).
– *Benefits of hearing aids* (in short: *benefits*), *Hearing aid stigma* (in short: *stigma*), *Sound quality and cost of hearing aids*, *Social pressure to get a hearing aid* (in short: *social pressure*), and *Evaluation of hearing aids by others* were measured using subscales of the Attitude Questionnaire (AQ; Van den Brink, 1995). The AQ was developed based on the HBM and validated on older Dutch hearing-impaired individuals (Van den Brink, 1995). *Benefits* included expected benefits of hearing aid use and were measured using the 10-item Benefits subscale. Scores can range from 10 to 50 (higher scores indicate greater benefit). *Stigma* was measured using the six-item Stigma subscale. Scores can range from 6 to 30 (higher scores indicate greater stigma). Expected barriers with regard to *sound quality* and *cost of hearing aids* were measured using the three-item Sound subscale. Scores can range from 3 to 15 (higher scores indicate more expected barriers). *Social pressure* was measured using the five-item Social Pressure subscale. Scores can range from 5 to 25 (higher scores indicate greater social pressure). The *evaluation of hearing aids by others* was measured using the three-item Evaluation of Aid subscale*.* The scale measures the respondent’s perception of others’ evaluation of the pros and cons of hearing aids in general (two items), and whether the respondent thought others would discourage him or her to get a hearing aid (one item). Scores can range from 3 to 15 (higher scores indicate more negative evaluation of hearing aids by others). Four AQ subscales showed a reasonable to good internal consistency in our study sample. Cronbach’s αs were .89 (Benefits), .83 (Stigma), .82 (Social Pressure), and .67 (Evaluation of Aid). The Sound subscale showed an unacceptable, low internal consistency (Cronbach’s α = .18) and was therefore excluded from the analyses.

#### Novel predictors

– *Passive acceptance of hearing loss* was measured by the two-item AQ subscale Passive Acceptance (Van den Brink, 1995). It refers to the perceived lack of need to do something about hearing problems in old age. Scores can range from 2 to 10 (higher scores indicate greater passive acceptance). The Spearman–Brown coefficient of this scale was 0.27 in our sample.
– *Precontemplation*, *Contemplation*, and *Action* were measured using the three subscales of the University of Rhode Island Change Assessment of the same name (URICA; McConnaughy, Prochaska, & Velicer, 1983). Each subscale consists of eight items and refers to a separate stage of change. The scores on each subscale can range from 8 to 40 (higher scores indicate a higher weight on the particular stage). The URICA was translated and validated for a Dutch nonclinical adult population by De Jonge, Schaap, and Schippers (2002). Items in the original URICA refer to “the problem,” which can be replaced by a specific health problem. For the purposes of this study, these items were adapted for hearing health behaviors by two of the authors (MP and SK) following the method applied by Laplante-Lévesque et al. (2013) for the English URICA. Laplante-Lévesque et al. (2013) showed good psychometric properties for all the URICA subscales in an older audiological population. The Precontemplation, Contemplation, and Action subscales showed reasonable to good internal consistency within our study sample. Cronbach’s αs were .68, .67, and .85, respectively.
– *Readiness for change* (in short: *readiness*) was determined by calculating the readiness composite score. This was done by adding a participant’s Contemplation and Action scores and subtracting his or her Precontemplation score (see Laplante-Lévesque et al., 2013). Scores could range from −24 (lowest readiness) to +72 (highest readiness).
– *Speech-in-noise recognition* was measured using the digit triplet speech-in-noise test administered over the telephone (Smits, Kapteyn, & Houtgast, 2004). The test determines an individual’s speech-reception threshold in noise (SRT_n_) defined as the signal-to-noise ratio (SNR) in dB SNR corresponding to 50% intelligibility. It correlates highly (*r* = .87) with the standard Dutch sentences speech-in-noise test (Plomp & Mimpen, 1979; Smits et al., 2004) and has shown satisfactory test–retest reliability in a sample of older participants (intraclass correlation coefficient = .70; Nachtegaal et al., 2009).
– *Age at onset of hearing problems* was measured by the item “At what age did you first notice your hearing problems? When I was … years old.”
*Maladaptive behavior*, *Verbal strategies*, *Nonverbal strategies*, *Self-acceptance*, *Acceptance of loss*, and *Stress and withdrawal* included coping behaviors and were measured using the six subscales of the 35-item Dutch short form Communication Profile for the Hearing Impaired (CPHI; Mokkink, Knol, Van Nispen, & Kramer, 2010). The six subscales reflect the degree of particular hearing coping behaviors that a person applies (i.e., use of communication strategies and personal adjustment). The CPHI was validated on Dutch hearing-impaired adults and has good psychometric properties (Mokkink et al., 2010). The Maladaptive Behavior subscale has seven items and scores can range from 7 to 35 (higher scores indicate less use of maladaptive behaviors such as avoiding conversations). The Verbal Strategies subscale has seven items and scores can range from 7 to 35 (higher scores indicate more use of verbal strategies such as asking for repetition). The Nonverbal Strategies subscale has five items and scores can range from 5 to 25 (higher scores indicate more use of nonverbal strategies such as positioning in a room). The Self-Acceptance subscale has four items and scores can range from 4 to 20 (higher scores indicate more self-acceptance of oneself with hearing loss). The Acceptance of Loss subscale has three items and scores can range from 3 to 15 (higher scores indicate more acceptance of hearing loss). The Stress and Withdrawal subscale has nine items and scores can range from 9 to 45 (higher scores indicate less stress and social withdrawal). All CPHI subscales showed acceptable to excellent internal consistency in our study sample. Cronbach’s αs were .78 (Maladaptive Behavior), .76 (Verbal Strategies), .82 (Nonverbal Strategies), .73 (Self-Acceptance), .72 (Acceptance of Loss), and .91 (Stress and Withdrawal).
*Self-efficacy of hearing aid handling* (in short: *self-efficacy*) was measured using the seven-item Basic Hearing Aid Handling subscale of the Measure of Audiologic Rehabilitation Self-Efficacy for Hearing Aids (MARS-HA). It possesses good psychometric properties (West & Smith, 2007). Scores can range from 0 to 100 (higher scores indicate greater self-efficacy). The questionnaire was translated from English to Dutch for the purposes of this study using the forward-backward method (Beaton, Bombardier, Guillemin, & Ferraz, 2000). Six persons were involved in this process. Excellent internal consistency was found in our study sample (Cronbach’s α = .96).
*Prompting consultation* referred to the form of the primary prompt for the initial visit to the HAD or ENT. It was defined as (a) self-initiated or encouraged by family, (b) referred or directed by another health-care professional (general practitioner or Audiological Center or ENT specialist or HAD), or (c) prompted by the HAD (i.e., advertisement: postal or media invitation).
*Agreement or discrepancy in views between the participant and the health-care professional about the necessity of a hearing aid* (in short: *Agreement or discrepancy*) was measured using an item that was based on excerpts collected in a study by Laplante-Lévesque et al. (2012). Responses were collected for the following statement: “My health care provider and I both think I need a hearing aid.” Response categories were “no, (s)he thinks I need a hearing aid, but I do not”; “yes, we both think I need a hearing aid”; “no, we both think I do not need a hearing aid”; “no, (s)he thinks I do not need a hearing aid, but I do”; “I do not know what (s)he thinks about the benefit of a hearing aid for me”; and “I don’t find any HCP’s opinion important. I know best how severe my hearing problems are.”
*Level of education* (highest level completed) was categorized into: low (uncompleted elementary, elementary, lower vocational), medium (general intermediate, intermediate vocational, general secondary, higher vocational), and high (college and university).
*Hours of paid work* were categorized into 0 hours per week, 1 to 20 hours per week, or 21 hours or over per week.
*Living situation* was defined as currently living with other people in the household or not.
*Country of birth* was dichotomized into the Netherlands or other.
*Social network size* (family and friendship networks) was measured using the six-item Lubben’s Social Network Scale, which has shown good psychometric properties in older European community*-*dwelling adults (Lubben et al., 2006)*.* Scores can range from 0 to 30 (higher scores indicate larger social networks). The scale showed good internal consistency in our study sample (Cronbach’s α = .86).
*Social participation* was measured using the nine-item Maastricht Social Participation Profile, which has been validated in older Dutch persons with chronic conditions (Mars et al., 2009). It measures frequency and diversity of consumptive (e.g., eating out) and formal social participation (e.g., organized day outings). Total scores can range from 0 to 27 (higher scores indicate higher levels of social participation). The scale showed reasonable internal consistency in our study sample (Cronbach’s α = .68).
*Personal computer (pc) use* was dichotomized into using a pc (desktop or laptop or palmtop or iPad or tablet) or not.
*Comorbidity of chronic diseases or conditions* (in short: *comorbidity*) was measured using the question: “Besides your hearing problems and possible vision problems or osteoarthritis, do you have any *other* longstanding illnesses or conditions?” Response options were *no* or *yes*. This item was based on an item that is used in the EU-Statistics on Income and Living Conditions instrument (Eurostat, 2013).
*Self-rated health* was measured using the question “How is your health in general?” (Van Sonsbeek, 1991). Response options were *very good* (0), *good* (1), *fair* (2), *sometimes good, sometimes poor* (3), or *poor* (4).
*Osteoarthritis of the hands* was assessed using the question “Do you suffer from osteoarthritis (degenerative arthritis) in your hands?” Response options were *no* or *yes*.
*Vision status* was measured using two items (near- and far-sighted vision) of the Organisation for Economic Co-operation and Development disability indicator (McWhinnie, 1979). The summed score can range from 0 to 6 (worst to best vision). The Spearman–Brown coefficient of this scale was 0.42 in our sample.
*Mastery* was measured by the abbreviated, five-item version of the Pearlin Mastery Scale (Pearlin & Schooler, 1978). The Pearlin Mastery Scale has shown good psychometric properties in adult samples (Pearlin & Schooler, 1978; Eklund, Erlandson, & Hagel, 2012). Scores can range from 5 to 25 (higher scores indicate a higher sense of mastery). The scale showed good internal consistency in our study sample (Cronbach’s α = .82).
*Cognitive dysfunction* was measured by the 20-item Cognitive Dysfunction Questionnaire (Vestergren, Rönnlund, Nyberg, & Nilsson, 2012). Scores could range from 20 to 100 (higher scores indicate higher cognitive dysfunction). The questionnaire was translated from Swedish into Dutch using the forward-backward method (Beaton et al., 2000). Five persons were involved in this process. Excellent internal consistency was found in our study sample (Cronbach’s α = .90).

### Candidate Effect Modifiers

*Gender*, *age*, and *readiness for change* were regarded as candidate effect modifiers. *Age* and *readiness for change* were analyzed as continuous variables.

### Statistical Analyses

#### Descriptive analyses

For each scale, the internal consistency was calculated using the full study sample. The Cronbach’s α was calculated for scales with three or more items and the Spearman–Brown coefficient for scales with two items (Eisinga, Grotenhuis, & Pelzer, 2013). Mean values and standard deviations (*SD*s), stratified by HAEP status, were calculated for continuous candidate predictors that followed a normal distribution. For continuous candidate predictors that followed a skewed distribution, medians and 25th and 75th percentile points were calculated. For dichotomous or categorical candidate predictors, as well as for the effect modifier *gender* and the corrective factor *source of recruitment* (defined later), proportions across HAEP status were calculated. Further, we tested the univariate associations between HAEP status and each of candidate predictors, *gender* and *source of recruitment*.

#### Collinearity analysis

Collinearity between candidate predictors was examined by calculating correlations and the predictors’ variance inflation factors for multicollinearity. This was done both for the final and for the starting prediction model before the backward selection procedure (see Prediction modeling section). The highest correlation found was .50 (between *benefits* and *social pressure*). All variance inflation factor values were well below 5, indicating no relevant collinearity.

#### Prediction modeling

Logistic multivariable regression models were built. Odds ratios (ORs) for each predictor were determined. When the OR was larger than 1, the OR indicated the increase in odds to enter a HAEP for 1 point increase in the predictor score. When the OR was smaller than 1, the OR indicated the decrease in odds for 1 point increase in the predictor score. Five steps were followed to build the models which are described later. A two-step approach (Steps 1 and 2) was chosen to take into account the previously obtained evidence on ‘likely’ predictors and build further on this evidence base. The variable *source of recruitment* was kept in the model in each step (forced) to adjust all predictors for this factor. Three sources of recruitment were defined: via an ENT department, via a HAD (persons who had an intake appointment scheduled), or via a HAD (persons who had no intake appointment scheduled). The adjustment for this factor was done because the different sources showed different univariate associations with HAEP status (see Table 2).

*Step 1*: *Determination of the basic model*. In this step, it was evaluated whether the seven ‘likely’ predictors were significant predictors of HAEP status. This was done by including the predictors in the starting model and performing a backward selection of variables using a *p_removal_* of .157. With this criterion, there is less optimism in regression coefficients as compared with a more strict α of .05 (Moons et al., 2015). The α of .157 corresponds to the use of the AIC for predictor selection, which is a method that accounts for model fit while penalizing for the number of parameters being estimated (see Moons et al., 2015).
*Step 2: Determination of the extended model*. In this step, it was evaluated whether ‘novel’ predictors significantly added predictive value to the basic model. This was done by creating reclassiﬁcation tables (which stratify individuals into risk categories of high risk or low risk of showing the outcome in question) and examining changes in categorization under a new model. Twenty-nine new models were created by separately adding the 29 ‘novel’ candidate predictors to the basic model. By evaluating the statistical significance (*p* < .05) of the Net Reclassification Improvement (NRI; Steyerberg et al., 2010), it was determined whether participants whose HAEP status were wrongly predicted by the basic model shifted to the correct outcome when a ‘novel’ predictor was added to the model. The NRI reflects the net percentage of improved classification (percentage improved minus percentage worsened). A threshold of 66% was used to distinguish between low and high risk in the reclassification tables because this was the prevalence of entering a HAEP within our study sample (see Descriptives subsection). The prevalence of the outcome can be used as a threshold when no clinically well-accepted threshold is available (Steyerberg et al., 2012). When more than one ‘novel’ predictor showed statistically significant added predictive value, the strongest ‘novel’ predictor (i.e., the one with the highest NRI) was added to the basic model first. Then, the second strongest predictor was added, after which the new NRI was tested. This procedure was repeated for all significant ‘novel’ predictors.
*Step 3*: *Testing effect modification*. Effect modification by *gender*, *age*, and *readiness* was tested for each of the predictor variables that was included in the extended model. This was done by separately including an interaction term for each of the predictor variables (e.g., *benefits* × *gender*) and determining its statistical significance (*p* < .05).
*Step 4*: *Determination of model performance.* The variance explained by the final prediction model was determined by calculating the Nagelkerke *R*^2^. The discriminatory power was determined by assessing the area under the curve of the receiver operator characteristic.
*Step 5*: *Determination of internal validity*. The final prediction model was internally validated using bootstrapping. This procedure results in an adjusted linear predictor, which is presented. The adjusted linear predictor includes the predictors’ effect sizes adjusted for the model’s shrinkage factor (Moons et al., 2015). Internal validation via preshrinkage is appropriate because prognostic models usually perform better in the patients who were used to build the model than in new patients, due to optimism in regression coefficients and performance measures (Moons et al., 2015). The bootstrapping procedure was carried out using the statistical software package R. For all other analyses, SPSS Statistics version 22 was used.

## Results

### Descriptives

Of the full sample (*n* = 377), 129 (34%) participants did not enter a HAEP at 4 months’ follow-up against 248 (66%) who did. The mean *age* of participants was 72.6 years (*SD* = 8.0), and 60% were men. Their *level of education* was reported as low (*n* = 90, 24%), medium (*n* = 238, 63%), and high (*n* = 48, 13%). *Hearing loss severity* ranged from 10 to 68 dB HL (mean: 39.8, *SD* = 9.1) and SRT_ns_ ranged from −7.4 dB SNR to +5.0 dB SNR. Table 1 shows the distribution of the participants across the candidate predictor variables and effect modifiers and across the *sources of recruitment*. Univariate associations are displayed in Table 2. Except for *age* and *stigma*, significant univariate associations with entering a HAEP were found for all ‘likely’ predictors (higher odds were associated with greater *hearing loss severity*, greater *self-reported hearing disability*, more *benefits*, greater *social pressure*, and more positive *evaluation of hearing aids by others*). Some of the ‘novel’ predictors also showed a significant association with HAEP status (see Table 2).

**Table 1.** Characteristics of the Study Sample (*n* = 377).

		*n* = 377			
Candidate predictors and effect modifiers (possible range)		Did not enter a hearing aid evaluation period (*n* = 129)		Entered a hearing aid evaluation period (*n* = 248)	
	mv	*n* (%^*^)	Mean (*SD*) or median [25th; 75th percentile]	*n* (%^*^)	Mean (*SD*) or median [25th; 75th percentile]
Likely predictors					
Age^⋄^	0	129	72.7 (8.2)	248	72.5 (8.3)
Hearing loss severity, 3F-BEA	0	129	37.1 (8.6)	248	41.2 (9.0)
Self-reported hearing disability (0–74)	15	121	21.1 (12.8)	241	31.7 (14.2)
Benefits (10–50)	8	128	34.4 (5.7)	241	40.0 (5.0)
Stigma (6–30)	1	129	13.7 (4.7)	247	14.3 (5.0)
Social pressure (5–25)	5	129	14.4 (3.8)	248	17.8 (3.4)
Evaluation of hearing aids by others (3–15)	10	127	7.7 (1.9)	240	7.1 (2.1)
Novel predictors					
Passive acceptance (2–10)	6	128	4.0 (1.3)	243	3.9 (1.4)
Precontemplation (8–40)	16	127	17.7 (5.4)	234	15.4 (5.4)
Contemplation (8–40)	17	127	27.9 (5.5)	233	31.4 (4.9)
Action (8–40)	14	125	26.6 (7.3)	238	32.4 (5.5)
Readiness^◊^ (−8 to 70)	24	125	37.1 (15.5)	228	48.4 (11.1)
Speech-in-noise recognition, SRT_n_	17	124	−3.3 (2.5)	236	−2.7 (2.6)
Age of onset of hearing problems	11	118	65 [56.5–72.0]	248	65 [58.0–70.0]
Maladaptive behavior (7–35)	1	129	33.0 [31.0–34.0]	247	31.0 [31.0–34.0]
Verbal strategies (7–35)	0	129	14.4 (4.0)	248	17.2 (5.2)
Nonverbal strategies (5–25)	1	129	14.5 (5.1)	247	15.8 (4.9)
Self-acceptance (4–20)	0	129	17.3 (2.9)	248	16.6 (3.1)
Acceptance of loss (4–15)	11	128	11.5 (2.6)	238	10.6 (2.6)
Stress and withdrawal (9–45)	3	128	36.0 (7.0)	246	32.5 (7.1)
Self-efficacy of hearing aid handling (0–100)	5	127	67.0 [55.0–70.0]	245	67.0 [57.5–70.0]
Prompting consultation	4				
self-initiated or encouraged by family		97 (44%)	–	123 (56%)	
referred by another HCP/prompted by the HAD		29 (19%)		124 (81%)	–
Agreement/ discrepancy	22				
R does not know what HCP thinks		37 (58%)	–	27 (42%)	–
disagreement—HCP: HA necessary; P: HA not necessary		16 (64%)	–	9 (36%)	–
disagreement—HCP: HA not necessary; P: HA necessary		8 (62%)	–	5 (38%)	–
agreement—HCP & P: HA necessary		26 (12%)	–	187 (88%)	–
agreement—HCP & P: HA not necessary		23 (85%)	–	4 (15%)	–
R only finds own beliefs about HA needs important		7 (50%)		7 (50%)	
Level of education	1				
low		27 (30%)	–	63 (70%)	–
medium		82 (35%)	–	156 (65%)	–
high		19 (40%)	–	29 (60%)	–
Hours of paid work	5				
0 hours per week		110 (35%)	–	204 (65%)	–
1–20 hours per week		12 (52%)	–	11 (48%)	–
21 hours or over per week		7 (20%)	–	28 (80%)	–
Living situation					
alone in the household	4	39 (38%)	–	63 (62%)	–
with others in the household		87 (32%)	–	184 (68%)	–
Country of birth					
The Netherlands		120 (34%)	–	236 (66%)	–
other country	1	8 (40%)	–	12 (60%)	–
Social network size (0–30)	5	287	17.8 (10.0)	240	16.6 (8.8)
Social participation (0–21)	8	125	6.7 (4.3)	244	6.2 (4.0)
Pc use					
no	1	25 (36%)	–	44 (64%)	–
yes		103 (34%)	–	204 (66%)	–
Comorbidity					
no	7	76 (35%)	–	141 (65%)	–
yes		51 (33%)	–	102 (67%)	–
Self-rated health (0–4)	4	127	1.1 (0.7)	246	1.2 (0.8)
Osteoarthritis in hands					
no	5	88 (33%)	–	180 (67%)	–
yes		38 (37%)	–	66 (63%)	–
Vision status (0–6)	2	127	6.0 [5.0–6.0]	248	6.0 [5.0–6.0]
Mastery (5–25)	6	127	10.2 (3.8)	244	10.4 (4.0)
Cognitive dysfunction (20–100)	7	127	30.5 (8.2)	248	31.3 (8.5)
Effect modifier^◊^					
Gender	0				
men		70 (31%)	–	155 (69%)	–
women		59 (63%)	–	93 (37%)	–
Corrective factor					
Source of recruitment	0				
ENT		25 (23%)	–	85 (77%)	–
HAD—intake appointment planned at baseline		22 (14%)	–	132 (86%)	–
HAD—no intake appointment planned at baseline		82 (73%)	–	31 (27%)	–

◊ Age and readiness were tested both predictors and effect modifiers. Gender was analyzed as an effect modifier only.

* Percentages only apply to dichotomous and categorical variables. Per category of the particular the variable (e.g., separately for men and women), the percentages indicate the proportion (of men/women) that did not enter a HEAP (left column), and the proportion (of men/women) that did enter a HAEP (right column).

SRT_n_: speech-reception threshold in noise in dB signal-to-noise ratio; ENT: ear nose and throat specialist; HAD: hearing aid dispenser; HCP: health care practitioner; HA: hearing aid; 3F-BEA: better-ear, pure tone average hearing threshold (dB HL) across 1, 2, and 4 kHz; mv: total number of missing values for this variable; P: participant; |: or; –: not applicable.

**Table 2.** Univariate Associations (Odds Ratios).

	Odds to enter a hearing aid evaluation period*		
Candidate predictors and effect modifiers (possible range / reference category)	OR	95% CI	*p*
Likely predictors			
Age^◊^	1.00	0.97–1.02	0.847
Hearing loss severity, 3F-BEA	**1.05**	**1.03–1.08**	**<0.001**
Self-reported hearing disability (0–74)	**1.06**	**1.04–1.08**	**<0.001**
Benefits (10–50)	**1.22**	**1.16–1.28**	**<0.001**
Stigma (6–30)	1.03	0.98–1.07	0.258
Social pressure (5–25)	**1.30**	**1.21–1.40**	**<0.001**
Evaluation of hearing aids by others (3–15)	0.86	**0.77–0.96**	**0.008**
Novel predictors			
Passive acceptance (2–10)	0.91	0.78–1.06	0.241
Precontemplation (8–40)	**0.93**	**0.89–0.96**	**<0.001**
Contemplation (8–40)	**1.14**	**1.09–1.19**	**<0.001**
Action (8–40)	**1.15**	**1.11–1.20**	**<0.001**
Readiness ^◊^ (−24 to +72)	**1.07**	**1.05–1.09**	**<0.001**
Speech-in-noise recognition, SRT_n_	**1.10**	**1.00–1.20**	**0.042**
Age of onset of hearing problems	1.00	0.98–1.01	0.796
Maladaptive behavior (7–35)	**0.93**	**0.88–0.99**	**0.029**
Verbal strategies (7–35)	**1.14**	**1.08–1.20**	**<0.001**
Nonverbal strategies (5–25)	**1.05**	**1.01–1.10**	**0.020**
Self-acceptance (4–20)	**0.93**	**0.86–1.00**	**0.059**
Acceptance of loss (4–15)	**0.87**	**0.80–0.95**	**0.002**
Stress and withdrawal (9–45)	**0.93**	**0.90–0.96**	**<0.001**
Self-efficacy of hearing aid handling (0–100)			
Prompting consultation (reference = self-initiated or encouraged by fam.) referred by another HCP/prompted by the HAD	3.37	2.08–5.47	**<0.001**
Agreement/ discrepancy (reference = R does not know what HCP thinks)			**<0.001**
disagreement—HCP: HA necessary; P: HA not necessary	0.77	0.30–2.00	0.593
disagreement—HCP: HA not necessary; P: HA necessary	0.86	0.25–2.91	0.804
agreement—HCP & P: HA necessary	**9.86**	**5.18–18.76**	**<0.001**
agreement—HCP & P: HA not necessary	**0.24**	**0.07–0.77**	**0.016**
R only finds own beliefs about HA needs important	1.37	0.43–4.37	0.594
Level of education (reference = low)			0.516
medium	0.82	0.48–1.38	0.445
high	0.65	0.31–1.36	0.257
Hours of paid work (reference = 0 hours per week)			**0.046**
1–20 hours per week	**0.49**	**0.21–1.16**	**0.104**
21 hours or over per week	**2.16**	**0.91–5.10**	**0.080**
Living situation—with others in the household (reference = alone)	1.31	0.82–2.10	0.265
Country of birth – other country (reference = the Netherlands)	0.76	0.30–1.92	0.564
Social network size (0–30)	1.02	0.98–1.05	0.395
Social participation (0–27)	0.97	0.92–1.02	0.273
Pc use (reference = no pc use)	1.13	0.65–1.94	0.671
Comorbidity (reference = no comorbidity)	1.08	0.70–1.67	0.736
Self-rated health (0–4)	**1.28**	**0.94–1.74**	**0.118**
Osteoarthritis of the hands (reference = no osteoarthritis)	0.85	0.53–1.36	0.498
Vision status (0–6)	0.90	0.70–1.15	0.384
Mastery (5–25)	1.01	0.96–1.07	0.664
Cognitive dysfunction (20–100)	1.01	0.99–1.04	0.385
Effect modifier^◊^			
Women^◊^ (reference = men)	**0.71**	**0.46–1.10**	**0.123**
Corrective factor			
Source of recruitment (reference = ENT)			**<0.001**
HAD—intake appointment planned at baseline	**1.76**	**0.94–3.33**	**0.079**
HAD—no intake appointment planned at baseline	**0.11**	**0.06–0.20**	**<0.001**

* Entered a hearing aid evaluation period = 1; did not enter a hearing aid evaluation period = 0 (reference category).

◊ Age and readiness were tested both as a predictor and an effect modifier. Gender was analyzed as an effect modifier only.

**Bold:** Statistically significant (*p* < 0.157).

SRT_n_: speech-reception threshold in noise in dB signal-to-noise ratio; ENT: ear nose and throat specialist; HAD: hearing aid dispenser; HCP: health care practitioner; HA: hearing aid; 3F-BEA: better-ear, pure tone average hearing threshold (dB HL) across 1, 2, and 4 kHz; P: participant; OR: odds ratio; CI: confidence interval; fam.: family; reference: reference category. For each of the candidate predictors, effect modifiers, and for source of recruitment, the odds to enter a hearing aid evaluation period is indicated.

### Prediction of Entering a Hearing Aid Evaluation Period

*Step 1*: *Determination of the basic model*. Five out of the possible seven ‘likely’ predictors constituted the basic model following backward selection. *Age* (*p = *.461) and *evaluation of hearing aids by others* (*p = *.306) were subsequently excluded from the model, as such leaving the following significant predictors: *benefits* (*OR* = 1.17, CI = 1.10-1.26], *p* < .001), *social pressure* (OR = 1.15, CI = 1.03–1.28, *p* = .010), *hearing loss severity* (OR = 1.04, CI = 1.00–1.08, *p* = .044), *self-reported hearing disability* (OR = 1.02, CI = 1.00–1.05, *p* = .076), and *stigma* (OR = 1.07, CI = 1.00–1.14, *p* = .048).
*Step 2: Determination of the extended model*. Four out of the 29 ‘novel’ predictors showed a statistically significant (*p*-values < .157) association with HAEP status after adding them to the basic model. However, their NRIs were small and nonsignificant, indicating that none of them significantly added predictive value to the model (NRIs: *agreement or discrepancy*: 3%, *prompting consultation*: 1%, *comorbidity*: 4%, *vision status*: 1%, *action*: 1%). To illustrate this further: *Comorbidity* showed an OR of 1.67 (CI = 0.87–3.24, *p* = .126) when it was added to the basic model but showed a nonsignificant NRI of 4%. For the remaining 25 ‘novel’ predictors, no significant association with HAEP status was found, nor were their NRIs significant. In conclusion, an extended model was not applicable.
*Step 3*: *Testing effect modification (by gender, age, readiness)*. No significant interaction by *age* was found (*p*-values of all interaction terms ≥.567). Significant interaction by *gender* was found for two predictors: for *hearing loss severity* (OR_Hearing Loss Severity×Gender_ = 1.09, CI = 1.01–1.18, *p = *.027) and for *stigma* (OR_Stigma×Gender_ = 1.16, CI = 1.01–1.32, *p = *.030). Table 3 displays the final gender-specific prediction model. Greater *hearing loss severity* was significantly predictive of entering a HAEP in women (*p = *.005) but not in men (OR = 1.01, CI = 0.97–1.06, *p = *.618). For women, the odds to enter a HAEP were 1.10 times (CI = 1.03–1.17) greater for each 1-dB increase in *hearing loss severity*. Likewise, greater *stigma* was significantly predictive of entering a HAEP in women (*p = *.015) but not in men (OR = 0.99, CI = 0.91–1.08, *p = *.836). For women, the odds to enter a HAEP were 1.13 times (CI = 1.03–1.26) greater for each 1-point increase on the stigma scale. For both men and women, higher odds to enter a HAEP were significantly associated with more expected *benefits* (OR = 1.19, CI = 1.11–1.27, *p* < .001), greater *social pressure* (OR = 1.15, CI = 1.03–1.28, *p* = .011), and greater *self-reported hearing disability* (OR = 1.03; CI = 1.00–1.05, *p* = .066).

**Table 3.** Final Multivariable Prediction Model Including the Modifying Effect by Gender of the Predictors Severity of Hearing Loss and Stigma. Odds Ratios for These Predictors are Presented Separately for Men and Women.

*n* = 351^◊^		Odds to enter a hearing aid evaluation period*		
Predictor (possible range / reference category)		OR	95% CI	**p**
Source of recruitment (reference category = ENT)		–	–	**<0.001**
HAD—intake appointment planned at baseline		**2.23**	**1.00–4.98**	**0.052**
HAD—no intake appointment planned at baseline		**0.18**	**0.09–0.39**	**<0.001**
Benefits (10–50)		**1.19**	**1.11–1.27**	**<0.001**
Social pressure (5–25)		**1.15**	**1.03–1.28**	**0.011**
Hearing loss severity, 3F-BEA_men_		1.01	0.97–1.06	0.618
Hearing loss severity, 3F-BEA_women_		**1.10**	**1.03–1.17**	**0.005**
Self-reported hearing disability (0–74)		**1.03**	**1.00–1.05**	**0.066**
Stigma_men_ (6–30)		0.99	0.91–1.08	0.836
Stigma_women_ (6–30)		**1.13**	**1.03–1.26**	**0.015**
*Explained variance:*	*(Nagelkerke R^2^)*	0.59		
*Calibration:*	*(Hosmer-Lemeshow test; p; H_0_ = good fit)*	*p* = 0.694		
*Discrimination:*	*(AUC of ROC; 95% CI)*	0.90	(0.87–0.94)	

ENT: ear, nose, and throat specialist; HAD: hearing aid dispenser; 3F-BEA: better-ear, pure tone average hearing threshold dB HL across 1, 2, and 4 kHz; OR: odds ratio; CI: confidence interval; AUC: area under the curve; ROC: receiver operator characteristic. Intercept of model: Beta = −8.43.

* Entered a hearing aid evaluation period = 1; did not enter a hearing aid evaluation period = 0 (reference category).

◊ Sample size does not equal 377 due to missing values.

**Bold**: Statistically significant (*p* < 0.157).

Adjusted linear predictor after bootstrapping: Intercept of model: Beta = −7.41, OR_HAD – f.u. appointment planned_ = 2.03, OR_HAD – no f.u. appointment planned_ = 0.22, OR_Benefits_ = 1.16, OR_Social pressure_ = 1.13, OR_3F-BEA men_ = 1.01, OR_3F-BEA women_ = 1.09, OR_Self-reported hearing disability_ = 1.02, OR_Stigma men_ = 0.99, OR_Stigma women_ = 1.12. Nagelkerke R^2 ^= 0.54; AUC = 0.89.

Significant interaction by *readiness for change* was found for one predictor: *self-reported hearing disability* (see Table 4). The OR of *self-reported hearing disability* was 0.92 (CI = 0.84–1.00, *p* = .059) and that of the interaction between *readiness* and *self-reported hearing disability* was 1.002 (CI = 1.000–1.004, *p* = .015). This indicated that for persons with relatively low readiness (*readiness* score ≤ 42), an increase in *self-reported disability* predicted decreasing odds to enter a HAEP (because the OR*_self-reported disability_* < 1), and this effect became somewhat weaker with increasing levels of readiness (because the OR*_self-reported disability_* then approached 1). At the same time, this result indicated that for persons with relatively high readiness (*readiness* score > 42), there was a positive predictive effect of *self-reported disability* (because for them the OR*_self-reported disability_ >* 1), and this effect became somewhat stronger with increasing levels of readiness. So for the latter group, greater severity of *self-reported hearing disability* predicted higher odds to enter a HAEP, with this effect being stronger for persons who were more ready for change.

**Table 4.** Final Multivariable Prediction Model Including the Modifying Effect by Readiness of the Predictor Self-Rated Hearing Disability.

*n* = 334^◊^		Odds to enter a hearing aid evaluation period*		
Predictor (possible range / reference category)		OR	95% CI	*p*
Source of recruitment (reference category = ENT)		**–**	**–**	**<.001**
HAD – intake appointment planned at baseline		**2.35**	**1.04–5.31**	**.040**
HAD – no intake appointment planned at baseline		**0.17**	**0.08–0.30**	**<.001**
Benefits (10–50)		**1.18**	**1.09–1.27**	**<.001**
Social pressure (5–25)		**1.13**	**1.01–1.26**	**.026**
Hearing loss severity, 3F-BEA		**1.05**	**1.01–1.09**	**.017**
Self-reported hearing disability (0–74)		**0.92**	**0.84–1.00**	**.059**
Self-reported hearing disability × Readiness		**1.002**	**1.00–1.00**	**.015**
Readiness		**0.96**	**0.91–1.01**	**.114**
Stigma (6–29)		**1.09**	**1.01–1.16**	**.018**
*Explained Variance:*	*(Nagelkerke R^2^)*	0.60		
*Calibration:*	*(Hosmer–Lemeshow test; p; H_0_ = good fit)*	*p* = .318		
*Discrimination:*	*(AUC of ROC; 95% CI)*	0.90	(0.87–0.94)	

ENT: Ear Nose and Throat specialist; HAD: hearing aid dispenser; 3F-BEA: better-ear, pure tone average hearing threshold (dB HL) across 1, 2, and 4 kHz; OR: odds ratio; CI: confidence interval; AUC: area under the curve; ROC: receiver operator c haracteristic. Intercept of model: Beta = −8.76. *Entered a hearing aid evaluation period = 1; did not enter a hearing aid evaluation period = 0 (reference category).

◊ Sample size does not equal 377 due to missing values. **Bold**: Statistically significant (*p* < .157).

Adjusted linear predictor after bootstrapping: Intercept of model: Beta = −7.98, OR_HAD – f.u. appointment planned_ = 2.18, OR_HAD – no f.u. appointment planned_ = 0.20, OR_Benefits_ = 1.16, OR_Social pressure_ = 1.12, OR_3F-BEA_ = 1.04, OR_Self-reported hearing disability_ = 0.93, OR_Self-reported hearing disability*Readiness_ = 1.002, OR_Readiness_ = 0.96, OR_Stigma_ = 1.08, Nagelkerke *R*^2 ^= 0.56; AUC = 0.89.

For the remaining four significant predictors, there was no interaction by *readiness*, so their predictive effects were independent of readiness (see Table 4). Those who expected more *benefits of hearing aids* (OR = 1.18, CI = 1.09–1.27, *p* < .001), experienced greater *social pressure to get a hearing aid* (OR = 1.13, CI = 1.01–1.26, *p* = .020) or experienced greater *stigma* (OR = 1.09, CI = 1.01–1.17, *p* = .020) had significantly higher odds to enter a HAEP.

*Step 4*: *Determination of model performance.* The variances explained by the gender-specific model (Table 3) and the readiness-specific model (Table 4) were similar and quite high (Nagelkerke *R*^2 ^= 0.59 and 0.60, respectively). This was also the case for the models’ discriminatory power (area under the curve = 0.90 for both models).

*Step 5*: *Determination of internal validity*. The adjusted linear predictors of the models are presented at the bottom of Tables 3 and 4, respectively. Similar shrinkage factors were found for the gender-specific and the readiness-specific models (0.89 and 0.91, respectively). These factors were reasonably high, thus indicating that the original models had reasonably good internal validity.

## Discussion

The current study aimed to identify variables that drive entering a HAEP. This is an important step in the hearing help-seeking journey of older persons with hearing problems in health-care systems in which an evaluation period is part of the hearing aid purchase process, like in The Netherlands. We built on established predictors of hearing aid uptake, and used the HBM and TTM as theoretical frameworks to select candidate predictors and to examine effect modification by *age*, *gender*, and *readiness for change*. Across all study participants, significant predictors of entering a HAEP were more expected *benefits of hearing aids* and greater *social pressure to get a hearing aid*. Greater experienced *hearing aid stigma* and greater *hearing loss severity* were modified by *gender* such that they were only predictive of entering a HAEP for women and not for men. Greater *self-reported hearing disability* was modified by *readiness for change* such that its positive predictive effect was stronger for persons who were more ready to do something about their hearing than for persons who were less ready. No modifications by *age* were found.

The fact that self-reported hearing disability, hearing loss severity, and social pressure emerged as significant predictors is not surprising as several studies have shown their relevance for driving other help-seeking steps (i.e., seeking professional help and taking up a hearing aid; Knudsen et al., 2010; Meyer et al., 2014; Van den Brink et al., 1996). With regard to self-reported hearing disability and hearing loss severity, Saunders et al. (2016a) and Meyer et al. (2014) only found greater hearing loss severity (4F-BEA) and not self-reported hearing disability to predict hearing aid uptake.

In contrast, in the current study, both hearing loss severity and self-reported hearing disability explained unique variability in the models, presumably by increasing the exposure to perceived threat of hearing loss (see HBM, Figure 1). Moreover, the more ready a person was, the more this person’s self-reported hearing disability predicted his or her likelihood of entering a HAEP. This is a novel finding. Although it is known that more advanced stages of change are associated with greater self-reported hearing disability, hearing loss severity, and hearing loss duration (Laplante-Lévesque et al., 2013; Laplante-Lévesque, Brännström, Ingo, Andersson, & Lunner, 2015), the present study is the first to indicate that readiness acts as an effect modifier of self-reported hearing disability. It should be noted that the model also indicated that within individuals who did not feel ready, the predictive effect of self-reported hearing disability was in the unexpected direction: Greater self-reported hearing disability predicted *lower* odds to enter a HAEP (and this effect became weaker with higher readiness). We do not know whether this effect is real or an artifact of the statistical model. In the former case, it may be speculated that the participants who did not feel ready may have been persons with very poor acceptance of their hearing loss who coped with this by turning away from hearing aids and thus from entering a HAEP (and they did this more strongly with increasing severity of hearing disability). However, then one would expect that correcting for the coping behaviors Acceptance of loss and Self-acceptance would weaken or nullify the effect. We performed this explorative analysis and this was not the case: The effect of self-reported hearing disability remained unchanged. Replication of the results in other studies should elucidate this further.

An important next step would be to identify the factors causing low readiness for change so that they can be influenced favorably as part of the help-seeking journey. In our view, such studies should take into account—but at the same time go beyond—factors that were previously identified as correlates of readiness (e.g., severity and duration of hearing loss and self-reported disability). Such factors can provide valuable information on target groups for intervention (e.g., persons with relatively mild hearing losses and short duration of complaints), but they do not necessarily reveal the true underlying mechanisms that cause low readiness. From an intervention perspective, the modifiable factors within these mechanisms then seem especially relevant. Researchers and hearing health-care professionals may be guided by the processes of change that the TTM describes in that respect (e.g., consciousness raising and self-reevaluation). According to the TTM, different types of processes should be emphasized during the successive stages of change (Babeu, Kricos, & Lesner, 2004; Ekberg, Grenness, & Hickson, 2016).

Surprisingly, the barrier of strong perceived hearing aid stigma did not predict low odds to enter a HAEP. Rather, for women, we found that greater perceived stigma predicted a *greater* likelihood of entering a HAEP. Further inspection of the data showed that the stigma scores of women who did not enter a HAEP were low (mean = 12.7), as compared with women who did enter a HAEP (mean = 14.8), men who did not enter a HAEP (mean = 14.3), and men who did enter a HAEP (mean = 14.1). It is possible that women who did not enter a HAEP unconsciously or consciously anticipated their choice not to enter a HAEP, and therefore either did not give stigma much thought or felt that stigma did not apply to them, in contrast to men who did not enter a HAEP. The gender difference we found may be surprising in the light of Garstecki and Erler’s (1998) results. They found that male nonadherents (who rejected professional advice to obtain hearing aids) found hearing aids more stigmatizing than male adherents (who followed such advice). This difference was not found in women. Replication of the current study’s findings is needed, as well as further research into the mechanism behind the counterintuitive relationship between stigma and HAEP status.

Another factor that emerged as a significant predictor in women, but not in men was better-ear severity of hearing loss. We do not have an explanation for this finding. Interestingly, Garstecki and Erler (1998) reported a similar result with respect to hearing aid uptake. Unfortunately, they did not explain this finding in their article. Further research is needed to elucidate this gender difference.

Although our final models showed reasonably high-explained variances, they did leave room for prediction by other factors (i.e., gender-specific model: *R*^2 ^= 0.59; readiness-specific model: *R*^2 ^= 0.60). The result showing that none of the examined ‘novel’ predictors added significantly to predictive strength was unexpected, as the reviews by Knudsen et al. (2010) and Meyer and Hickson (2012) had identified many of them as promising candidates.

In particular, for two of these promising candidates (cognition and self-efficacy of hearing aid handling), Meyer et al. (2014) showed that they significantly predicted hearing aid uptake. Meyer et al. (2014) found a significant predictive effect by one specific cognitive factor only (cognitive reasoning skills). We did not include this cognitive factor, but Meyer et al. (2014) emphasized that it explained very little variance, suggesting minor relevance. Based on the nonsignificant prediction by self-efficacy that was found in this study and the contrasting significant prediction by this factor found by Meyer et al. (2014), we hypothesize that expectations about one’s capacity to handle a hearing aid may be less relevant for the decision to enter a HAEP. This may be explained by the fact that an evaluation period in the Netherlands allows persons to try out a hearing aid without commitment and free of any costs. As such, expected poor handling skills may be considered harmless and no reason to forego the HAEP, whereas in hearing aid uptake, it is.

### Study Limitations

This study has some limitations that deserve discussion. First, previous studies showed that perceived barriers toward hearing aids, and, in particular, concerns over hearing aid cost, are key barriers to hearing aid uptake (Fischer et al., 2011; Garstecki & Erler, 1998; Kochkin, 2007; Laplante-Lévesque et al., 2012; Meyer et al., 2014). The reimbursement system in the Netherlands is quite complicated and cost of a hearing aid depends on a person’s health insurance coverage and the particular hearing aid category that the purchased hearing aid falls under. The assessment of the hearing aid category and the choice of the particular hearing aid would occur after the baseline measurement of the current study; and therefore, it was decided that *expectations* about cost was the only possible and sensible construct to measure as a predictor. However, the Sound subscale showed an unacceptably low-internal consistency so that it had to be excluded from the analyses. As such, the predictive value of the barriers of expected high cost and poor sound quality could not be assessed within this study. Van den Brink (1995) developed the AQ and found a much higher internal consistency for the Sound subscale (i.e., Cronbach’s α = .48, unpublished data). In contrast, Meyer et al. (2014) performed a factor analysis on the AQ and did not find that the items of the original Sound subscale formed a separate factor. Van den Brink’s (1995) and Meyer et al.’s (2014) samples partly consisted of hearing aid users, and this may explain why the Sound-items behaved differently in their samples as compared with the sample of the current study. Nonetheless, the results do show that the Sound subscale that we used was invalid, and a different scale should have been used to capture the barriers related to expected hearing aid sound quality and cost.

This study incorporated the HBM and the TTM. Both psychological models have received much criticism over the years, but this mainly concerned critiques expressed in other health behavior fields (see Coulson et al., 2016, for an overview). One major concern for the TTM has been the lack of convincing evidence of the existence of distinct stages of readiness and thus whether a progression from one stage to another actually takes place. Another is the possible instability of stage scores over time (people may change stages quickly). However, these critiques can be refuted for hearing help-seeking, as several studies have shown that the stages of change have satisfactory construct and concurrent validity and can predict help-seeking, intervention take-up, and intervention outcomes across periods of 6 to 18 months (e.g., Ingo et al., 2016; Laplante-Lévesque et al., 2013; Saunders et al., 2013, 2016a, 2016b).

Another much-cited critique of both the TTM and the HBM is that they rely on rational reasoning only, and unconscious influences on behavior are barely considered (see Coulson et al., 2016). Along similar lines, Saunders et al. (2016a) stated that automatic motivational processes such as emotions and habits may play an important role in hearing help-seeking and it may be worthwhile to use models that do take such factors into account. Heffernan, Coulson, Henshaw, Barry, and Ferguson (2016) suggested that Leventhal’s self-regulatory model would be such a model. We subscribe to these ideas.

### Conclusion, Clinical Implications, and Future Directions

This study aimed to comprehensively and prospectively examine the predictors of entering a HAEP in older persons who were eligible for a hearing aid. The results show that many well-known predictors of hearing aid uptake are also predictive of HAEP status. The HBM proved valuable in identifying factors reflecting the perceived threat of hearing loss (self-reported hearing disability, hearing loss severity, social pressure to get a hearing aid) and the perceived benefits of starting a HAEP (expected benefits of hearing aids). Furthermore, the current study led to the notion that the relevance of some of these predictors (hearing loss severity, stigma, and self-reported hearing disability) may depend on gender and the level of readiness for behavior change (TTM). As this was the first study examining predictors prospectively while applying effect modification analyses, we strongly advocate for future longitudinal studies to test the external validity of the models. In addition, we recommend researchers to explore the underlying mechanisms of some of the unexpected results we found (prediction by stigma and hearing loss severity in women).

After assuring external validity, an important next step would be to develop prediction rules that could be used in clinical practice. The factors that would cause a particular patient to be unlikely to enter a HAEP in the near future can then be identified early and subsequently addressed using counseling (mapping a person’s hearing problems, creating awareness, and acceptance of hearing loss, thereby possibly influencing self-reported hearing disability), education (for realistic expectations about hearing aid benefits), and involvement of significant others (to facilitate social support). As previously described, an important research gap to fill in this context would be the identification of the determinants of readiness for change (and their interplay), so that these can be targeted through intervention as well.
